# Supplementary material for: Assessing Burden, Anxiety, Depression, and Quality of Life among Caregivers of Hemodialysis Patients in Indonesia: A Cross-Sectional Study
Source: Int J Environ Res Public Health. 2022 Apr 9;19(8):4544. doi: 10.3390/ijerph19084544 (PMC9032362; doi:10.3390/ijerph19084544)
Supplement: Supplementary file 1 [file ijerph-19-04544-s001.zip › ijerph-1582139-supplementary.pdf]

Supplemental Figure S1 (1-6)

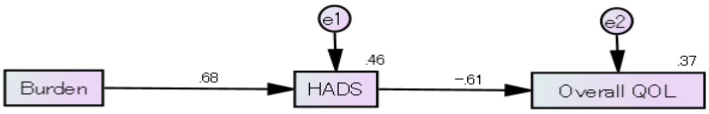

Figure S1-1. Path analysis among variables: Overall QOL as a dependent variable

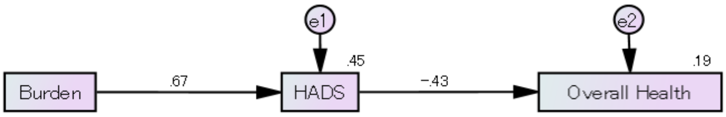

Figure S1-2. Path analysis among variables: Overall Health as a dependent variable

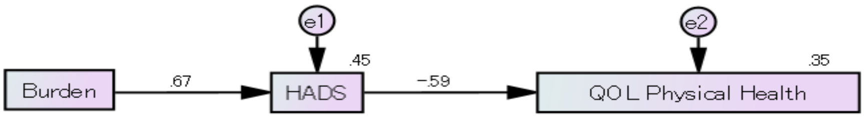

Figure S1-3. Path analysis among variables: Physical QOL as a dependent variable

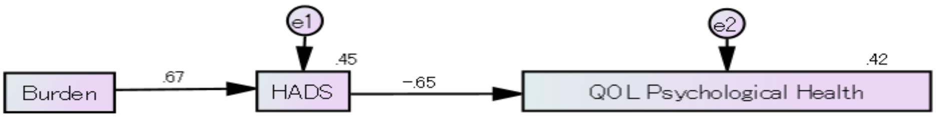

Figure S1-4. Path analysis among variables: Psychological QOL as a dependent variable

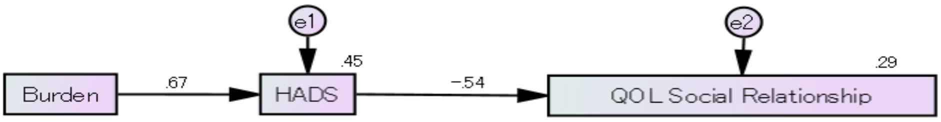

Figure S1-5. Path analysis among variables: Social relationship as a dependent variable

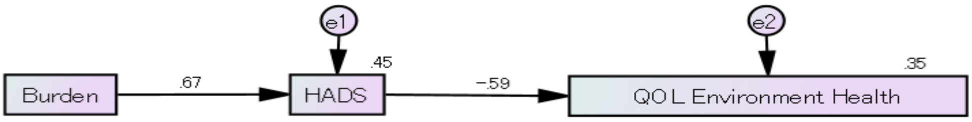

Figure S1-6. Path analysis among variables: Environment QOL as a dependent variable

Note. HADS: Hospital Anxiety and Depression Scale; QOL, Quality of Life
